# The role of lung ultrasound in the diagnosis of pneumonia in acutely ill patients

**DOI:** 10.1186/2036-7902-6-S2-A2

**Published:** 2014-08-27

**Authors:** M Biancardi, B D’Agostino, C Bonfardeci

**Affiliations:** 1 Medicina 1 dept, Ospedale San Carlo Borromeo, Milano, Italy; 2 Medicina d’ Urgenza, Ospedale San Carlo Borromeo, Milano, Italy

## Background

The differential diagnosis of patients in the emergency department who show symptoms of sepsis and suspected pneumonia may be difficult because of the low sensitivity and specificity of supine chest X rays (SCXR) and physical examination.

## Objective

A retrospective assessment of the usefulness of lung ultrasound (LU) in the diagnosis of pneumonia in patients with signs and symptoms of sepsis and respiratory disease.

## Patients and methods

2 groups of patients were considered:

Group A: 22 patients with a positive first SCXR (SCXR1) for pneumonia

Group B: 18 patients with a negative SCXR1 for pneumonia, but who were subsequently diagnosed with pneumonia during their hospital stay following a second SCXR (SCXR2) or CT.

Lung ultrasound examination was performed within 24 hours of admission, searching for direct and indirect echographical signs of pneumonia: focal B lines (FBL), pleural effusion (PE) and images of lung consolidation (LC).

## Results

See table 1.

**Table 1:** 

	Group A (%)	Group B (%)
Male/Female	11/11	7/11

MEAN AGE	74,8	76,8

SCXR1 pos	22	0

SCXR2 or CT pos	-	18

FBL pos	9 (41)	10 (50)

FBL neg	13 (59)	10 (50)

PE pos	17 (78)	16 (80)

PE neg	5(22)	4 (20)

LC pos	19 (87)	20(100)

LC neg	3 (13)	0 (0)

LC pos or FBL pos	21 (95)	-

LU impact on therapy	No	20 (100)

## Conclusions

In our observational study, LU showed high sensitivity in the diagnosis of pneumonia:

95% in patients already diagnosed using SCXR, and 100 % of early diagnosis of pneumonia in patients whose diagnosis was subsequently confirmed during hospitalization.

## References

[B1] Lichtenstein DA Relevance of Lung Ultrasound in the Diagnosis of Acute Respiratory Failure - The Blue Protocol Chest 2008 6 117 125 10.1378/chest.07-2800 18403664 PMC3734893

[B2] Essayag Y Diagnostic value of chest radiographs in bedridden patients suspected of having pneumonia Am J Med 2010 6 88. e 1 5 10.1016/j.amjmed.2009.09.012 20102999

[B3] Reissig A Sonographic Diagnosis and Follow-up of Pneumoniai: A Prospective Study Respiration 2007 6 537 547 10.1159/000100427 17337882

[B4] Mathis G Thoraxsonography – Part II: Peripheral Pulmonary Consolidation Ultrasound in Med. & Biol 1997 6 8 1141 1153 10.1016/S0301-5629(97)00111-7 9372562

[B5] Volpicelli G Detection of sonographic B lines in patients with normal lungs or radiographic alveolar consolidation Med Sci Monitor 2008 6 3 CR 122 128 18301355

